# Dysbiotic Oral and Gut Viromes in Untreated and Treated Rheumatoid Arthritis Patients

**DOI:** 10.1128/spectrum.00348-22

**Published:** 2022-08-30

**Authors:** Ruochun Guo, Shenghui Li, Yu Zhang, Yue Zhang, Guangyang Wang, Hayan Ullah, Yufang Ma, Qiulong Yan

**Affiliations:** a Department of Microbiology, College of Basic Medical Sciences, Dalian Medical University, Dalian, China; b Puensum Genetech Institute, Wuhan, China; c College of Food Science and Nutritional Engineering, China Agricultural University, Beijing, China; University of Georgia

**Keywords:** microbiome, community variation, oral and gut, rheumatoid arthritis, virome

## Abstract

Rheumatoid arthritis (RA) is influenced by oral and gut bacteria; however, much less is known about the relationship between oral or gut viromes and RA. Here, we performed whole-oral- and whole-gut-virome analyses based on shotgun sequencing of 497 samples. A comparative analysis of the oral and gut viromes in healthy controls and untreated and treated RA patients was performed, and system interaction networks among viruses, bacteria, and RA-associated clinical indices were constructed to address the potential relationship between the virome and RA by principal-coordinate analysis, distance-based redundancy analysis, permutational multivariate analysis, Spearman correlation coefficient analysis, and random-forest model analysis. The results showed that the viromes could be profiled in dental plaque, saliva, and fecal samples, among which saliva had the highest within-sample diversity. Importantly, significantly different diversities and compositions of the oral (i.e., dental plaque and saliva) viromes were observed not only between RA patients and healthy controls but also between untreated and treated RA patients, yet there were relatively minor differences in the gut viromes. Furthermore, to understand how these viruses affected the bacteriome, a virus-bacterium interaction network was constructed from dental plaque, saliva, and fecal samples of RA patients. Additionally, some RA-associated oral taxa, including *Lactococcus* phage (vOTU70), Bacteroides vulgatus, Lactococcus lactis, Escherichia coli, and Neisseria elongata, were correlated with the RA-related clinical indices. Whole-virome analysis illustrated the potential role of the oral and gut viromes in affecting our body either directly or via bacteria, which characterized neglected and new candidates contributing to the development of RA.

**IMPORTANCE** Our results demonstrated community variation among dental plaque, saliva, and fecal viromes. In oral and gut samples from untreated and treated RA patients, the perturbance of viral composition and the correlation network of microbes and RA-associated clinical indices might be involved in the pathogenicity of RA. The findings in this study expand the knowledge of the potential role of oral and gut viral communities in the development of RA and may contribute to research on correlations between viruses and other diseases.

## INTRODUCTION

Rheumatoid arthritis (RA) is a chronic autoimmune and inflammatory disease that is closely correlated with the homeostasis of the human microbiome (1–10). According to previous studies on gut microbiota, an enriched abundance of Prevotella copri and decreased abundances of *Bacteroides*, *Bifidobacterium*, and Eubacterium rectale were associated with untreated recent-onset RA (11). An opportunistic oral pathogen, *Porphyromonas gingivalis*, was frequently observed in association with pathogenesis of RA (12, 13). Other studies based on whole-metagenome shotgun sequencing revealed a significant enrichment of Lactobacillus salivarius and a reduction of Haemophilus in the gut as well as dental plaque and saliva samples of RA patients (2). Additionally, the abundances of several *Prevotella* and Streptococcus members in the human respiratory tract were also linked to RA (14).

As an important part of the whole microbiome, the viral community plays an well-recognized role in phenotypic and disease variations among individuals (15). Clooney et al. observed the changes in the gut viral composition with increased numbers of temperate bacteriophages in Crohn’s disease (16). Increased proportions of *Adenoviridae* sequences in the gut (17) and the cervical virome (18) were detected in human immunodeficiency virus (HIV)-infected patients. Even vaginal viruses were associated with bacterial vaginosis (BV), serving as an implicit factor in the perturbation of the bacterial community (19). Although previous studies found that microbial communities affect these diseases as well as RA, we knew little about the connection between RA and viromes. Notably, the potential impact of viruses on RA was widely hypothesized (20–23). One study indicated that respiratory viral infections might be a risk factor for the development of RA (20). Some studies have linked Epstein-Barr virus (EBV) to the pathogenesis of RA, with an abnormal increase in EBV-infected B cells in the blood, brain, and ectopic lymphoid structures of RA patients (21, 23–25). These findings showed that the virus may play a part in the development of RA.

Thus, in this study, we focused on the alterations of the viral community and ecological networks of bacteria and viruses in RA patients by whole-virome analysis. A data set from a previous study by Zhang et al. was reanalyzed (2), involving a total of 497 samples from three body sites (dental plaque, saliva, and feces) of 102 healthy controls (HCs), 104 untreated RA patients, and 60 treated RA patients. We characterized the viral population in human oral and gut communities and further assessed the potential perturbation of these viromes in untreated and treated RA patients. Meanwhile, we identified interaction networks among viruses, bacteria, and RA-associated clinical indices in this cohort.

(This article was submitted to an online preprint archive [26].)

## RESULTS

### Subjects.

This study involved a cohort of 266 individuals (164 RA patients and 102 healthy controls) from Shanghai, China. A total of 104 RA patients were new-onset patients without any medication treatment, while the other 60 patients were treated with multiple drugs. The phenotypic and clinical characteristics of the individuals were introduced in the preliminary study by Zhang et al. (2). A total of 44.7% (119/266) of individuals provided both fecal and oral (dental plaque and saliva) samples, while 34.2% (91/266) of the individuals provided all three samples (see Fig. S1A in the supplemental material). Finally, 143 dental plaque (51 healthy controls, 54 untreated patients, and 38 treated patients), 122 saliva (47 healthy controls, 51 untreated patients, and 24 treated patients), and 232 fecal (97 healthy controls, 94 untreated patients, and 41 treated patients) samples were analyzed (Fig. S1B). For each body site, the corresponding individuals’ gender, age, and body mass index (BMI) were matched and elaborated in the original study (Tables S1 and S2) (2).

### Viral populations in oral and gut communities.

To characterize the oral and gut viral communities, we analyzed a total of 2.72 Tbp of high-quality nonhuman metagenomic data (5.48 ± 1.77 Gbp per sample) from 497 dental plaque, saliva, and fecal samples. Metagenomic assemblies on each sample generated a total of 2,664,228 contigs (length of ≥5,000 bp) (Table S1), of which 6.1% (*n* = 161,828) were recognized as highly credible viral fragments based on their sequence features and homology to known viral genomes (see Materials and Methods). After removing the redundant viral contigs with 95% nucleotide similarity (27), totals of 19,483, 28,123, and 30,805 viral operational taxonomic units (vOTUs) were identified from the dental plaque, saliva, and gut metagenomes, respectively. To investigate the human virome across oral and gut samples, we combined the vOTUs from the three habitats and generated an integrated nonredundant catalog of 48,718 vOTUs for further analysis. Based on the assessment by CheckV (28), 15.5% of the vOTUs were high- or medium-quality viral genomes, and the remaining vOTUs were usually low-quality viral fragments or undetermined sequences (Fig. S2). A total of 55.1% of the vOTUs could be classified into known families. However, only 6% of the vOTUs manifested ≥95% nucleotide similarity with available sequenced viruses in the RefSeq database, highlighting the considerable novelty of the oral and gut virome data set.

**FIG 1:**
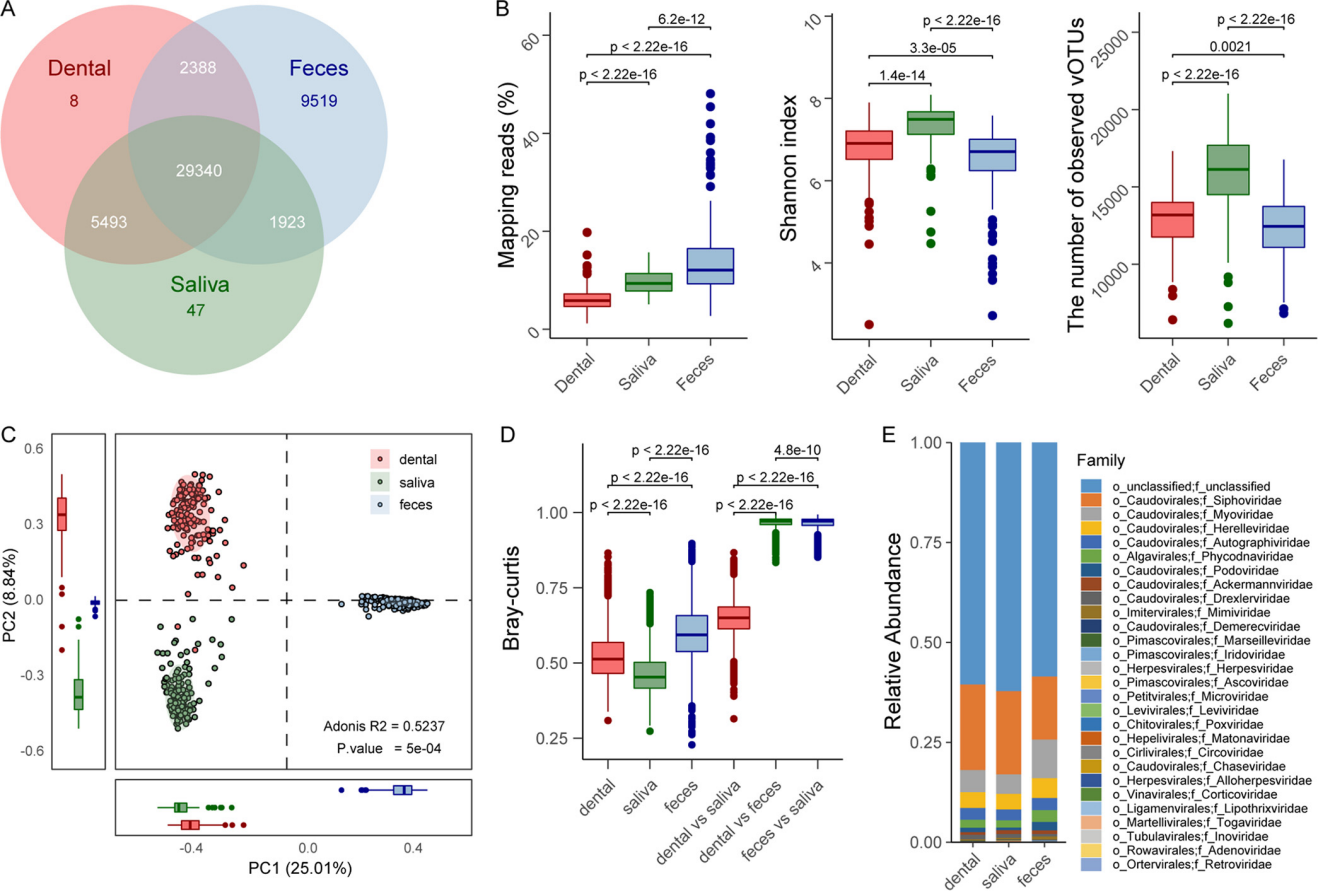
Overview of the oral and gut viromes. (A) Overlap of vOTUs among the dental plaque, saliva, and fecal viromes. (B) Comparison of the fractions of reads mapping to all vOTUs and the alpha diversity indices among the three habitats. (C) Principal-component analysis (PCA) based on the vOTU compositional profiles of samples from the three habitats. (D) Bray-Curtis distances among samples by body habitat. (E) Relative abundances of viral compositions at the family level for the three habitats. Significance was calculated using unpaired Wilcoxon’s rank sum test.

Totals of 37,229, 36,803, and 43,170 unique vOTUs were observed in 143 dental plaque, 122 saliva, and 232 gut metagenomes, respectively. The dental plaque and saliva metagenomes shared markedly high proportions (93.6% in dental plaque and 94.6% in saliva) of vOTUs between each other, and they both shared a relatively lower number of vOTUs with the gut virome (85.2% in dental plaque and 84.9% in saliva) (Fig. 1A). A total of 77.9% of the vOTUs of the gut virome were also detected in oral samples, and the remaining 22.1% of the vOTUs were gut specific. Focusing on the viral diversity in every sample from the three habitats, we found that the fecal samples had a significantly high proportion of viral sequences (on average, 14.0%) in their metagenome compared with those of the dental plaque (6.3%) and saliva (9.5%) samples (Fig. 1B, left). However, the saliva samples had higher within-sample diversity (estimated by the Shannon index) and richness (estimated by the number of observed vOTUs) than dental plaque and fecal samples, while these parameters were lowest in the fecal samples (Fig. 1B; Fig. S3).

Principal-component analysis (PCA) based on the vOTU composition revealed a clear separation among the 3 habitats (Fig. 1C). On the PCA plot, the gut virome was significantly stratified to the oral virome at the primary principal coordinate (PC1) (explaining 25.0% of the total variance), while the dental plaque and saliva viromes had deviated at the secondary principal coordinate (PC2) (explaining 8.8% of the total variance). Consistently, an intuitive comparison of Bray-Curtis dissimilarity indices showed that the gut viromes were quite far away from the dental plaque and saliva viromes (Fig. 1D). At the family level, all three habitats were dominated by viruses of five families (ignoring the family-level unclassified vOTUs): *Siphoviridae*, *Myoviridae*, *Herelleviridae*, *Autographiviridae*, and *Phycodnaviridae* (Fig. 1E; see Table S3 for the full list of viral families). The gut virome had a lower proportion of *Siphoviridae* and higher proportions of the other 4 families than the dental plaque and saliva samples.

### Oral and gut viromes are disturbed in untreated and treated RA patients.

A comparative analysis was conducted to investigate the differences in oral and gut viral communities among healthy controls and untreated and treated RA patients. Alpha and beta diversities were estimated based on the compositions of all vOTUs. To identify RA-associated biomarkers, multiple comparisons were performed based on 1,944 high-abundance vOTUs with a >0.01% mean relative abundance in all samples.

**FIG 2:**
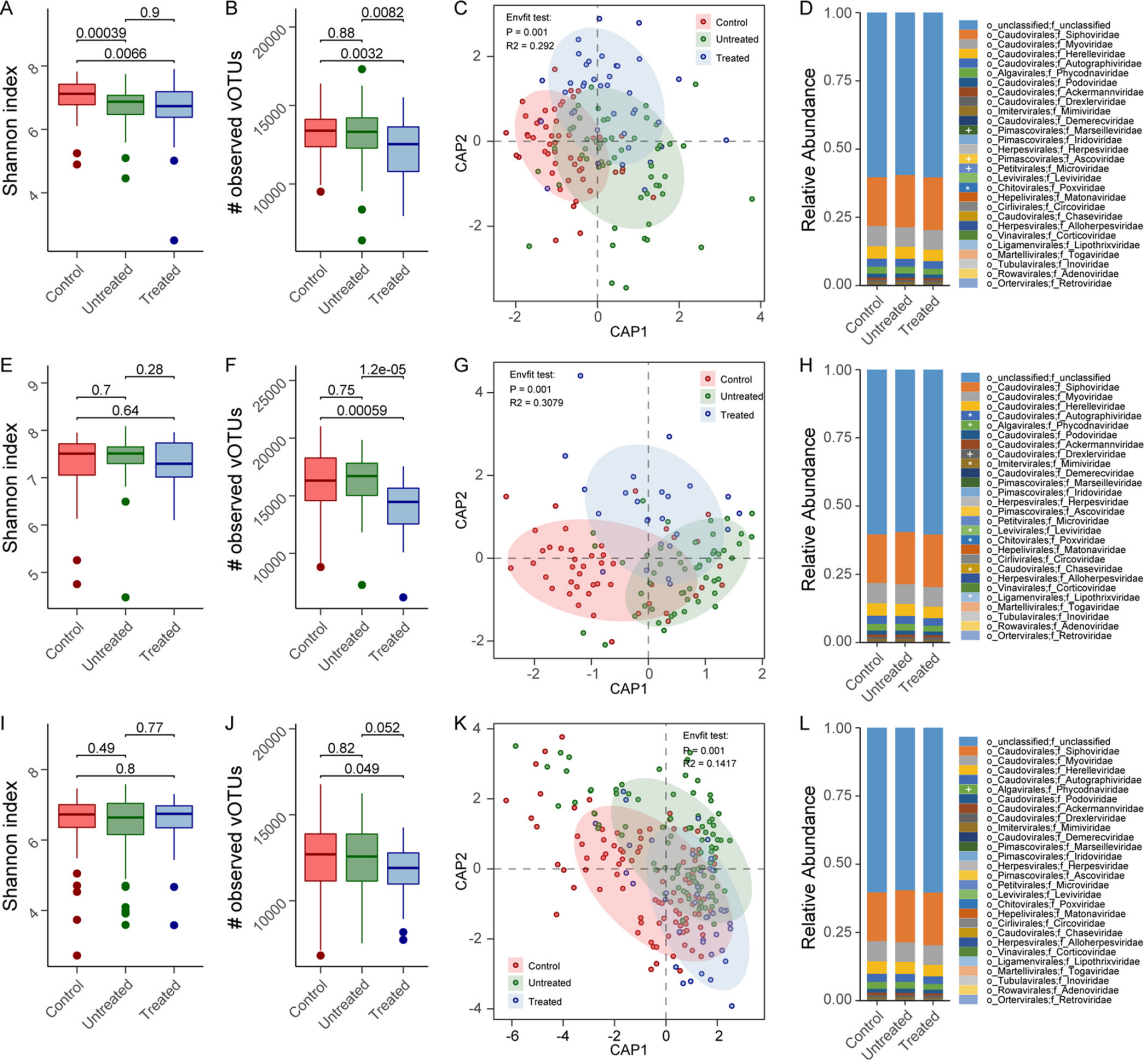
Community variation of the oral and gut viromes of RA patients. (A, B, E, F, I, and J) Alpha diversity indices among healthy controls and untreated and treated patients with RA (dental plaque [A and B], saliva [E and F], and fecal [I and J] samples). Significance was calculated using Wilcoxon’s rank sum test. (C, G, and K) Distance-based redundancy analysis (dbRDA) based on Bray-Curtis distance of the vOTU compositions in healthy controls and untreated and treated patients with RA (dental plaque [C], saliva [G], and fecal [K] samples). The x-axis and y-axis represent the first two constrained principal coordinates (CAP1 and CAP2). (D, H, and L) Family-level viral compositions in healthy controls and untreated and treated patients with RA (dental plaque [D], saliva [H], and fecal [L] samples). Significance was calculated using the Kruskal-Wallis test. +, *P *< 0.05; *, *P *< 0.01.

For the dental plaque virome, the untreated and treated RA patients had significantly lower Shannon indices than the healthy controls (*P* < 0.05 by a Wilcoxon rank sum test) (Fig. 2A). The viral richness values for untreated patients were close to those for the healthy controls (*P* > 0.05 by a Wilcoxon rank sum test), while those for the treated patients were still significantly lower than those for the other subjects (*P* < 0.05 by a Wilcoxon rank sum test) (Fig. 2B). Distance-based redundancy analysis (dbRDA) and envfit analysis revealed a visible deviation among the three cohorts (*R*^2^ = 29.2%; *P* = 0.001) (Fig. 2C). Fourteen of 679 highly abundant (>0.01% abundance in all dental plaque samples) vOTUs displayed significant differences in relative abundances among the three cohorts (adjusted *P* < 0.05 by a Wilcoxon rank sum test) (Table S4). These vOTUs included a *Lactococcus* phage (vOTU70) that was enriched in the dental plaque viromes of treated patients (Fig. S4A); this phenomenon is potentially linked to the usage of dairy products in some RA patients (29, 30). At the family level, the relative abundances of four families, *Marseilleviridae*, *Ascoviridae*, *Microviridae*, and *Poxviridae*, differed among the three cohorts (Fig. 2D; Table S5).

For the saliva virome, the Shannon indices for the three cohorts were equal, whereas the viral richness in treated patients was significantly lower than those in untreated patients and healthy controls (Fig. 2E and F). dbRDA and envfit analysis revealed visible deviations among the three cohorts (*R*^2^ = 30.8%; *P* = 0.001) (Fig. 2G). A total of 463 of 633 highly abundant vOTUs had significant differences in relative abundances among the three cohorts (adjusted *P* < 0.05 by a Wilcoxon rank sum test) (Table S6). Unlike the dental plaque virome, *Lactococcus* phage vOTU70 was enriched in the oral viromes of both healthy subjects and treated RA patients (Fig. S4B). At the family level, the relative abundances of 8 families, *Autographiviridae*, *Phycodnaviridae*, *Drexlerviridae*, *Mimiviridae*, *Leviviridae*, *Poxviridae*, *Chaseviridae*, and *Lipothrixviridae*, differed among the three cohorts (Fig. 2H; Table S5), of which a dominant viral family, *Autographiviridae*, was significantly enriched in the untreated patients.

For the gut virome, both the Shannon index and viral richness values were similar among the three cohorts (Fig. 2I and J). dbRDA and envfit analysis revealed visible deviations among the three cohorts (*R*^2^ = 14.2%; *P* = 0.001) (Fig. 2K), even though the effect size of disease states was relatively small for the gut virome (*R*^2^ = 14.2%) compared to those for the dental plaque and saliva viromes (*R*^2^ = 29.2% and 30.8%, respectively). Seventy-three of 975 highly abundant vOTUs had significant differences in relative abundances among the three cohorts (adjusted *P* < 0.05 by a Wilcoxon rank sum test) (Table S7). Besides, only the abundance of the family *Phycodnaviridae* differed among the three cohorts (Fig. 2L; Table S5), and it was significantly decreased in the gut viromes of treated RA patients.

Taken together, our findings revealed that the viral diversity, numbers of vOTUs, and family composition were remarkably altered in the oral viromes of untreated and treated RA patients, while these alterations occurred at a relatively low incidence in the gut viromes of RA patients.

### Interactions of viruses and bacteria are disrupted in treated RA patients.

The bacterial microbiome (as well as the archaeal microbiome, jointly referred to as the “bacteriome” here) of all samples was quantified by totaling 225 high abundant species (abundance of >0.05% in all samples) based on read mapping of unique clade-specific bacterial/archaeal marker genes (31). Thirty-five species (adjusted *P *< 0.05), 59 species (adjusted *P *< 0.05), and 6 species (adjusted *P *< 0.05) had differing relative abundances among the three habitats (Table S8).

**FIG 3:**
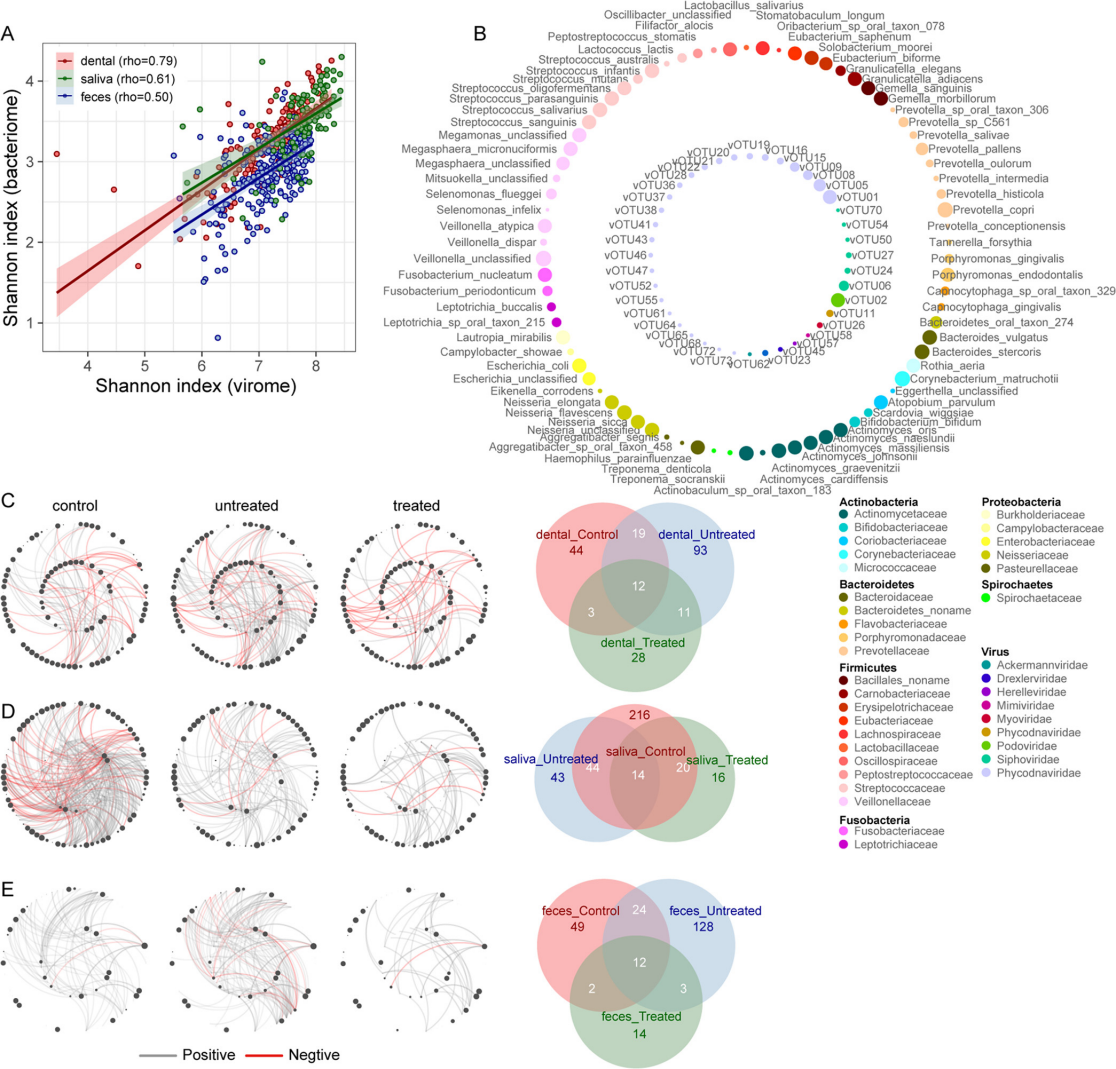
Correlation between the bacteriome and virome. (A) Correlation analysis of Shannon indices between the bacteriome and virome in the three habitats. (B to E) Correlation networks based on the relative abundances of bacteria and viruses with a >0.05% average relative abundance in all samples (dental plaque [C], saliva [D], and fecal [E] samples). In panel B, nodes in the inner and outer rings indicate vOTUs and bacteria, respectively. Nodes are colored according to taxonomic assignment at the family level. In panels C to E, red lines indicate a negative correlation, and gray lines indicate a positive correlation (adjusted *P *< 0.05 by a Spearman correlation test). Venn diagrams show the numbers of shared significant associations between highly abundant vOTUs and bacterial species in samples from healthy controls and untreated and treated RA patients.

Focusing on viral diversity, we found that the Shannon index was strongly correlated between the virome and bacteriome in all three ecosystems and all cohorts (Fig. 3A; Fig. S5), suggesting an extensive connection between viruses and bacteria. To study such interactions, we built coabundance networks between 75 high-abundance vOTUs and 225 bacterial species with an average abundance of >0.05% in all samples (Fig. 3B). In the dental plaque group, virus-bacterium associations were highest in the untreated RA patients, followed by the healthy controls, and RA patients were at the bottom (Fig. 3B). Furthermore, healthy controls and untreated RA patients were more closely associated with each other than with the treated RA patients. A similar result was also observed for the gut virome (Fig. 3D). In saliva, virus-bacterium associations appeared most frequently in the healthy control cohort, while the treated RA patients still showed a remarkable reduction (Fig. 3C). Overall, these findings revealed that the virus-bacterium interaction network is disrupted in treated RA patients.

### Oral viruses and bacteria are frequently linked to RA-associated clinical indices.

**FIG 4:**
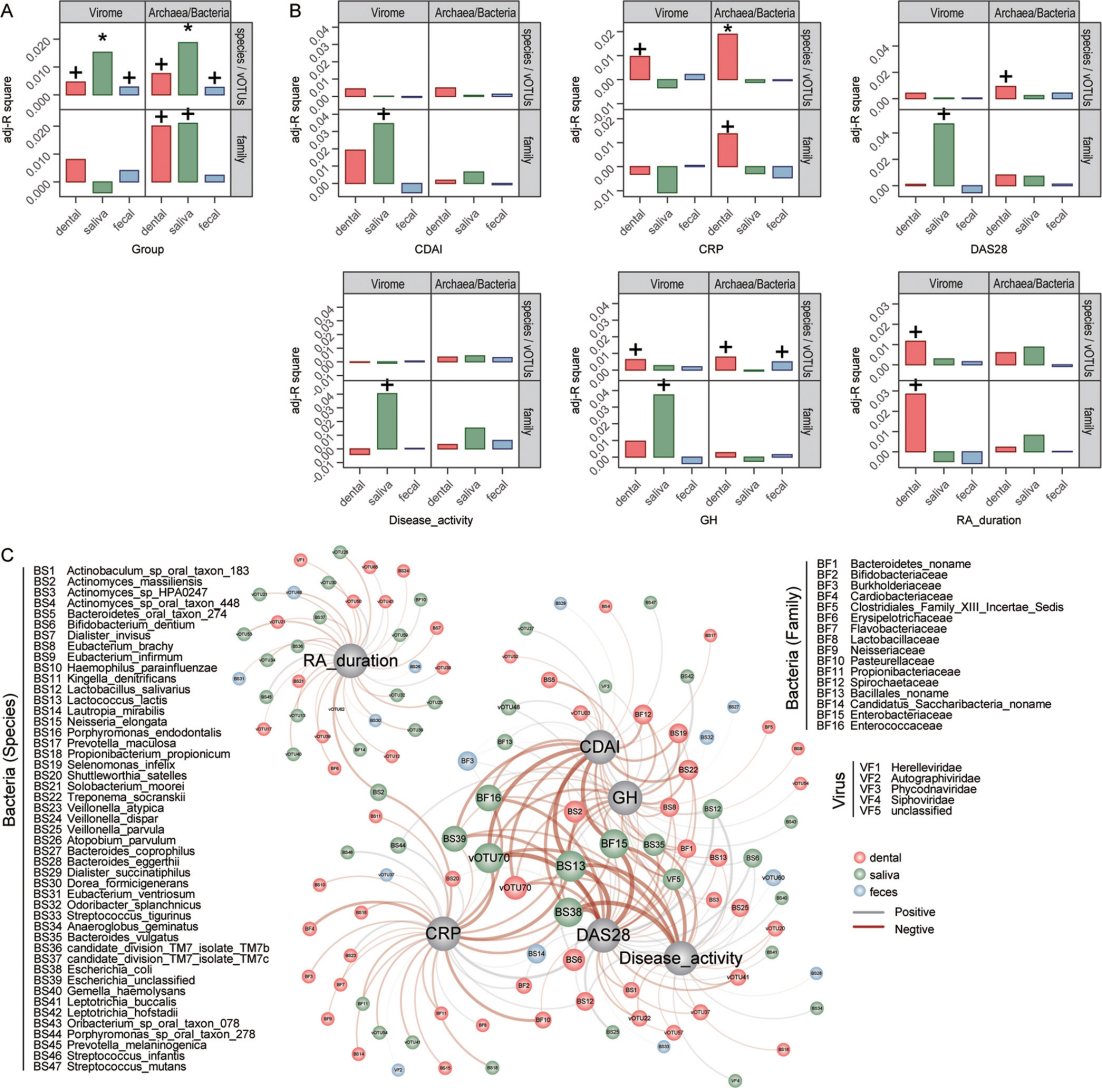
Correlation among the virome, bacterial microbiome, and clinical indices. (A) Effect size of RA status on the oral and gut viromes and bacteriomes (+, adjusted *P *< 0.05; *, adjusted *P *< 0.01 [by an Adonis test]). (B) Effect sizes of several RA-associated indices on the oral or gut virome. (C) Correlation network of 6 RA-associated clinical indices and microbes (adjusted *P *< 0.05 [by a Spearman correlation test]). The edge weight indicates the strength of the correlation. The node size (for microbes) indicates the relative abundance of taxa in different habitats.

We then wanted to investigate the effects of host characteristics and clinical parameters on the oral and gut viromes and bacteriomes. In accordance with the results of the above-mentioned analysis, the stratification of the three cohorts (untreated and treated RA patients and healthy controls) significantly affected both oral and gut viromes and bacteriomes at the vOTU/species level, and this influence was highest in the saliva virome and bacteriome but lowest in the gut virome and bacteriome (Fig. 4A). Of the host demographic variables, age and gender had a considerable impact on the dental plaque virome and bacteriome but were not associated with the gut virome, whereas BMI and weight were significantly associated with the gut virome and bacteriome (Fig. S6A). Of the clinical parameters, we found that several RA-associated indices were significantly associated with the oral virome. For example, C-reactive protein (CRP), general health (GH), and RA duration were associated with the dental plaque virome, while the clinical disease activity index (CDAI), disease activity score 28 (DAS28), disease activity, and GH were associated with the oral virome (Fig. 4B). Of the medications, methotrexate (MTX) indices were significantly associated with the gut bacteriome at the species level and the dental plaque bacteriome at the family level but were not associated with the oral and gut viromes (Fig. S6C). Besides, the oral virome was associated with several other clinical indices, such as the dental plaque virome being associated with hemoglobin (HGB) and the saliva virome being associated with platelet (PLT) and white blood cell (WBC) counts (Fig. S6B and D).

We generated a large correlation network of 6 RA-associated clinical indices and microbes (including both viruses and bacteria with a >0.05% average relative abundance in all samples) (Fig. 4C). All indices were correlated with a variety of bacteria and viruses from dental plaque and saliva, but only a relatively small number of gut microbes were involved. Several dental plaque microbes, including Actinomyces massiliensis, Treponema socranskii, Eubacterium brachy, and *Lactococcus* phage vOTU70, as well as several saliva microbes, including Lactococcus lactis, Bacteroides vulgatus, Escherichia coli, and *Lactococcus* phage vOTU70, were the keystone taxa in the network, which were associated mainly with CDAI, GH, CRP, DAS28, and disease activity. In summary, these findings revealed broad connections among oral viruses, bacteria, and RA-associated clinical indices.

### Prediction of RA status by employing viral and bacterial communities.

**FIG 5:**
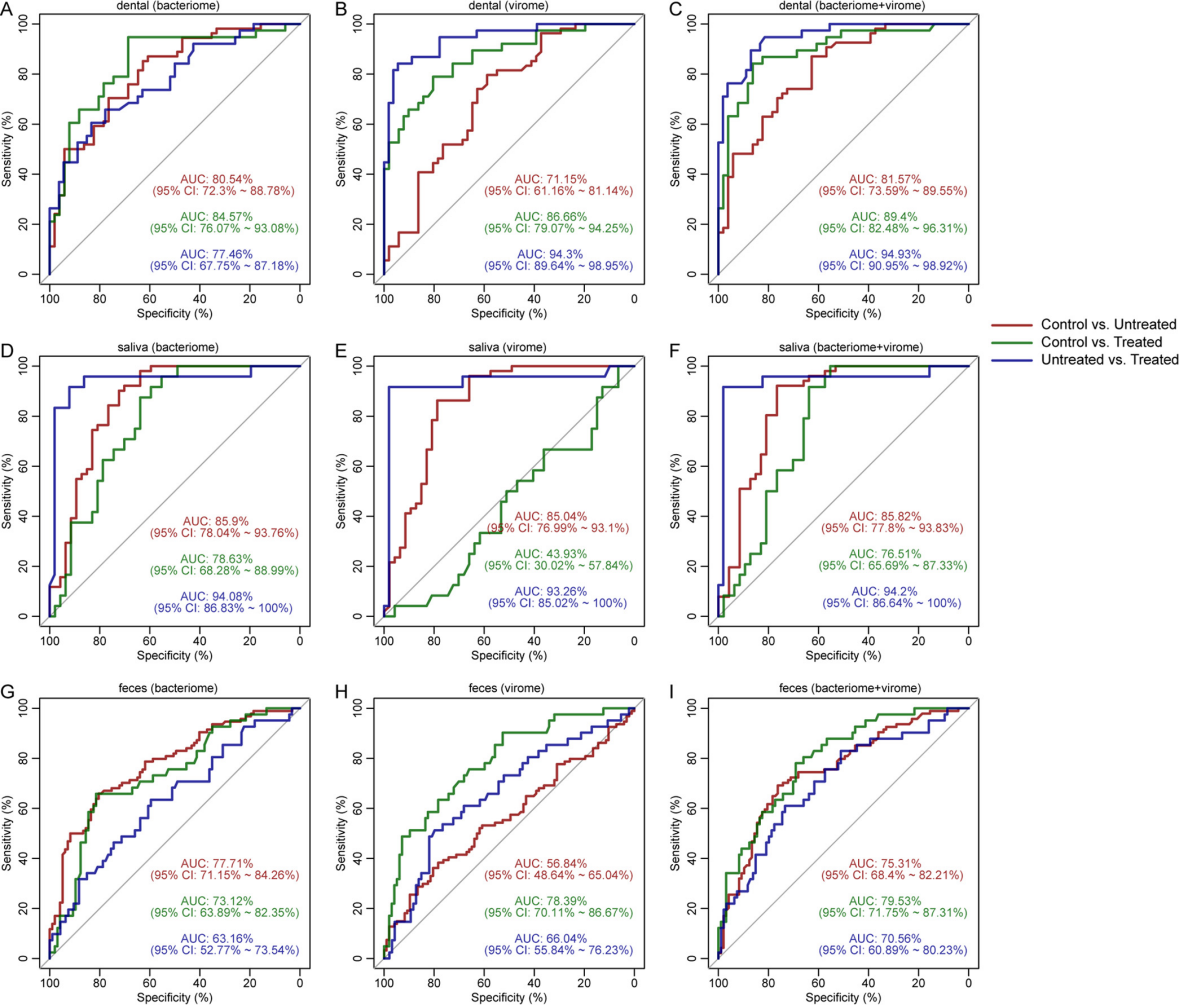
Classification of samples from healthy controls and untreated and treated patients with RA. RA-associated makers for fecal (A to C), dental plaque (D to F), and salivary (G to I) samples from healthy controls and untreated and treated RA patients were used.

Finally, we classified individuals into three statuses based on both the bacteriome and virome using the random-forest model. Each model featured highly abundant bacterial species and vOTUs (>0.05% relative abundances and significant differences). The performance of each of the models was assessed by the leave-one-out cross-validation method. For dental plaque, the bacteriome achieved areas under the curve (AUCs) of 80.54%, 84.57%, and 77.46% for the discrimination of untreated patients versus controls, treated patients versus controls, and untreated versus treated patients (Fig. 5A), respectively. For the dental plaque virome, the level of discrimination between untreated patients and controls was much lower (AUC = 71.15%) than that between untreated and treated patients (AUC = 94.3%) (Fig. 5B). Combining the bacteriome and virome achieved AUCs of 81.57%, 89.4%, and 94.93% for the discrimination of untreated patients versus controls, treated patients versus controls, and untreated versus treated patients (Fig. 5C), respectively. For the saliva bacteriome and virome, the AUCs were nearly equivalent to those for dental plaque, with a higher ability to discriminate untreated patients versus controls and a lower ability to discriminate treated patients versus controls (Fig. 5D to F). Inversely, for the gut bacteriome and virome, the AUCs were significantly lower than those for dental plaque and saliva (Fig. 5G to I). These results suggested that the microbes in dental plaque and saliva have a higher predictive power for RA status compared with those in feces.

## DISCUSSION

We reanalyzed a public data set from a previous RA microbiome research study (2). Using the 48,718 vOTUs identified, we discovered alterations in oral and gut viral diversity and composition as well as virus-bacterium relationships between untreated and treated RA patients. Furthermore, as several specific viruses have been reported to be associated with the etiology of autoimmune diseases (32, 33), we substantialized these viruses at the metagenome-based virome level.

Oral habitats (dental plaque and saliva) had a low proportion of virome sequences but high within-sample diversity (including Shannon’s diversity index and the number of observed vOTUs) compared with those of fecal samples, which was consistent with previous findings that the human oral cavity had fewer bacteria but high diversity (34–36). However, the contrast was that the oral and gut bacterial contents are rarely shared (<5% shared genes in a previous study by Tierney et al. [36] and <20% shared genes in the preliminary RA study [2]), whereas the oral and gut viromes highly overlapped, with >80% shared vOTUs. This suggested that the oral and gut viruses might transfer or communicate more frequently than bacteria. Further research is needed to clarify the potential mechanism of viral exchange between oral and gut viral communities. Notably, contrary to the within-sample diversity, oral habitats (dental plaque and saliva) had lower interindividual diversity of the viral community than fecal samples, which are characterized by high individual variability and a stable viral composition over time (37). Some studies reported that the individual variability of the gut microbiome drives susceptibility and resistance to host environmental changes (38–40), such as dietary intervention and infection. Although the oral virome showed a relatively low degree of interindividual variability, the oral virome was more sensitive than the viromes from other body sites in some cases, such as antibiotic therapy (41). These findings suggested that the oral virome should receive more attention in the future, particularly regarding its association with oral viruses and human diseases.

Compared to the gut virome, little is known about the association between the oral virome and human diseases. Interestingly, whether in untreated or treated RA patients, we found that the RA-associated changes of the oral virome were remarkably greater than those of the gut virome. For the oral virome, 57 dental plaque vOTUs and 463 saliva vOTUs had significantly different abundances between untreated/treated RA patients and healthy controls. One of these vOTUs, *Lactococcus* phage vOTU70, showed overgrowth in the dental plaque samples of treated RA patients and the saliva samples of both controls and treated patients. Although *Lactococcus* phages were rarely linked to human diseases, a previous study reported an increase in lytic *Lactococcus* phages in patients with Parkinson’s disease (42). Notably, a previous study linked several species of the bacterial genus *Lactococcus* to RA status (2). Our findings indicated that there was also a potential connection between *Lactococcus* phages and RA treatment. Given these variations in viral compositions between cohorts/groups, the oral virome was more sensitive to RA-associated clinical parameters. For example, the saliva virome could be significantly influenced by two parameters regarding RA disease activity (DAS28 and disease activity), suggesting its potential as a clinical indicator. Previous studies showed that the oral bacteriome deviates from a healthy status in RA patients (2, 43) and was associated with multiple clinical indices for RA such as the DAS28 score, CRP, and anti-cyclic citrullinated peptide (anti-CCP) antibody (2). These findings complied with recent opinions that RA patients’ oral health is related to their disease activity/progression (43). Meanwhile, the dental plaque virome was influenced by CRP and RA duration, suggesting that oral viruses play a role in both the short-term inflammation level and the long-term disease progression of RA patients.

Previous research focused on the impact of the gut virome on human health and disease. For example, patients with inflammatory bowel disease (IBD) had higher viral diversity and richness than healthy controls (16, 44). Similar results were observed in a study on colorectal cancer (45). In contrast, in this study, there was only a small difference in the alpha diversities of the HC and RA groups. Even so, the effect size of RA status on the gut virome was considerable (*R*^2^ = 14.2%), as 73 vOTUs were identified with different abundances among untreated and treated patients and controls in this study. Although a previous study reported similar viral community compositions in healthy controls and individuals at risk for RA, it revealed many RA-associated viral biomarkers (10). Moreover, the coaltered oral and gut viruses found in this study suggested a potential role of the oral-gut virome axis in the pathogenesis and treatment of RA, which was partly consistent with the results of recent studies proposing a role of the oral-gut microbiome axis in the etiology of RA (46, 47). Notably, some studies based on Epstein-Barr virus-targeted PCR reported that Epstein-Barr virus could cause RA by infecting synovial tissues (48, 49); however, this virus was not recovered from our oral and gut metagenomic data sets, possibly due to a low proportion of Epstein-Barr virus in the oral cavity and gut.

Close interactions of the gut virome and bacteriome have been reported for several immunity-associated diseases such as RA (2, 50), systemic lupus erythematosus (SLE) (50), multiple sclerosis (50), IBD (16, 51), and cirrhosis (52). However, so far, few studies have reported virus-bacterium interactions in the oral ecosystem. Here, we enriched their relationships in the oral ecosystem by constructing virus-bacterium coabundance networks in both dental plaque and saliva samples. Interestingly, we found that the virus-bacterium networks in both untreated and treated RA patients were remarkedly changed compared with those in healthy controls, and these changes were much more considerable in treated RA patients, with the manifestation of virus-bacterium associations decreasing at all three body sites. The alteration of virus-bacterium interactions may suggest a perturbance of the whole ecosystem (53), establishing a deeper connection with the pathogenicity of RA.

Finally, we explored the feasibility of combining bacteriome and virome data to distinguish RA and healthy control groups. The results showed that the AUC values could reach 89.4% for discriminating between controls and treated patients (dental plaque samples), 85.82% for discriminating between controls and untreated patients (saliva samples), and 94.2% for discriminating between treated and untreated patients (saliva samples). These AUC values were equal to or higher than those based on the bacteriome or virome alone, indicating that the model combining the bacteriome and virome can effectively distinguish RA status for early diagnosis, but systematic investigations of key viral markers might be helpful in the future. A similar predictable effect of the virome was also observed previously in the gut of IBD patients (16).

## MATERIALS AND METHODS

### Virus Identification and analyses.

**(i) Metagenomic data and assembly.** Metagenomic data for 497 samples (143 dental plaque, 122 saliva, and 232 fecal samples) from 266 individuals (164 RA patients and 102 healthy controls) were obtained from the NCBI database under BioProject accession no. PRJEB6997. Quality control for metagenomic reads was performed using fastp with the options -q 20 -u 30 -n 5 -y -Y 30 -l 90 –trim_poly_g -w 20 (54), and the human reads were removed based on a Bowtie2 alignment (55). The remaining reads were defined as high-quality nonhuman metagenomic data. Each sample was individually assembled using metaSPAdes (56). Proteins of the assembled contigs were predicted using Prodigal (57).

**(ii) Viral identification and vOTUs.** The assembled contigs (≥5,000 bp) were identified as viruses when they satisfied one of the following criteria: (i) at least 50% of the proteins of a contig (or at least 3 proteins if the contig had fewer than 6 proteins) were assigned to the integrated viral protein database from NCBI reference viral genomes and the Virus Orthologous Groups database (https://vogdb.org), with a maximum pairwise alignment E value of 1e−10 based on DIAMOND (58); (ii) the contigs were classified as belonging to virus categories 1, 2, 4, and 5 using VirSorter (59), a homology-based viral identifier using assembled metagenomic data; and (iii) the contigs had a score of >0.9 and a *P* value of <0.05 using VirFinder (60), a k-mer-based tool for identifying viral sequences. To reduce bacterial DNA contamination as much as possible, according to a previous study (61), we searched bacterial universal single-copy orthologs (BUSCO) (62) within viral contigs using hmmsearch and calculated the ratio of the number of BUSCOs to the total number of genes in each viral sequence (BUSCO ratio). Next, we removed highly contaminated viral contigs with a ≥5% BUSCO ratio, and the remaining contigs were considered the final viral contigs for each sample. Viral contigs were subjected to a pairwise BLAST analysis, and highly consistent viruses with 95% nucleotide identity and 80% coverage of the sequence were further clustered into vOTUs using in-house scripts. The longest viral contig was defined as the representative sequence for each vOTU. Finally, the genome quality of vOTUs was assessed by CheckV (28).

**(iii) Taxonomic classification.** Protein sequences of all vOTUs were first predicted using Prodigal with the option -p meta (57). Proteins of the vOTUs were aligned with the vConTACT2 reference (63) using BLASTP (minimum score of 50), and a family-level taxonomy of a vOTU was generated if more than one-third of its proteins were assigned to the same viral family (64).

**(iv) Taxonomic abundances.** To calculate the relative abundances of vOTUs, clean reads in each sample were first mapped against vOTU contigs using Bowtie2 alignment. Reads mapping to each vOTU were aggregated using SAMtools (65), leading to an average of 6.10 million viral reads per sample (for more details, see Table S1 in the supplemental material). The percentage of mapped reads is calculated as the total number of assigned reads divided by the number of high-quality nonhuman metagenomic reads. The relative abundance of each vOTU was calculated as the total number of assigned reads divided by its genome size and the total number of reads mapping to all vOTUs in each sample. The relative abundance profiles of viral families were generated by adding up the abundances of vOTUs annotated as belonging to the same family. In addition, to obtain the bacteriome composition, clean reads in each sample were mapped to clade-specific markers using MetaPhlAn2 (31).

### Bioinformatic and statistical analyses.

**(i) Alpha and beta diversities.** Rarefaction analysis was performed to estimate the sequencing depth using a custom script. We randomly subsampled the reads at a sequencing depth of 400 with intervals ranging from 10,000 to 4,000,000 reads, with 10 replicates for each interval. Alpha diversities in each sample were estimated based on the rarefaction data: (i) the number of observed OTUs was the number of vOTUs with a nonzero abundance, and (ii) the Shannon diversity index was calculated using the vegan diversity function. Beta diversity was evaluated using Bray-Curtis dissimilarity based on the relative abundance of all vOTUs using the vegdist function in the R vegan package.

**(ii) Multivariate statistics.** All statistical analyses and figure generation were carried out in R (v.4.0.2). Principal-component analysis (PCA) was performed based on the relative abundance profiles of vOTUs using the dudi.pca function from the R package ade4. Distance-based redundancy analysis (dbRDA) based on Bray-Curtis dissimilarity was carried out using the capscale function of the R package vegan, and the significance value was estimated based on 1,000 permutations by the envfit function. In addition, to evaluate the explained variation of the different covariates on the microbial composition, permutational multivariate analysis of variance (PERMANOVA) was performed using the adonis function. The effect size of a variable was determined as the *R*^2^ value adjusted using the RsquareAdj function, and a *P* value of <0.05 was considered significant.

**(iii) Prediction model.** Random-forest models were carried out based on vOTUs or bacterial species with significant differences among the disease groups, using the randomForest function. Receiver operating characteristic (ROC) curves with leave-one-out cross-validation were used to evaluate the model using the roc function of the R pROC package.

**(iv) Statistical test.** To determine the associations between taxa and covariates or between vOTUs and bacterial species, the Spearman correlation coefficient was measured using the cor.test function in R, which could be used to define the potential interactions between viruses and bacteria. Correlation networks were visualized using Cytoscape (66). Tests of significance between two groups for taxonomic abundances were performed using the wilcox.test function, while tests of significance among multiple groups were performed using the kw.test function. *P* values from the comparative analysis were adjusted using the fdrtool function. *P* values from correlation analysis were adjusted using the p.adjust function with the “method=BH” parameter. A *P* value of <0.05 was considered different, and an adjusted *P* value of <0.05 was considered significant.

### Data availability.

The relevant data and scripts had been submitted to GitHub at https://github.com/RChGO/RAvir/.
