# Supplementary material for: Dysbiotic Oral and Gut Viromes in Untreated and Treated Rheumatoid Arthritis Patients
Source: Microbiol Spectr. 2022 Aug 30;10(5):e00348-22. doi: 10.1128/spectrum.00348-22 (PMC9603985; doi:10.1128/spectrum.00348-22)
Supplement: Supplemental file 1 — Fig. S1 to S6. Download spectrum.00348-22-s0001.pdf, PDF file, 0.6 MB [file spectrum.00348-22-s0001.pdf]

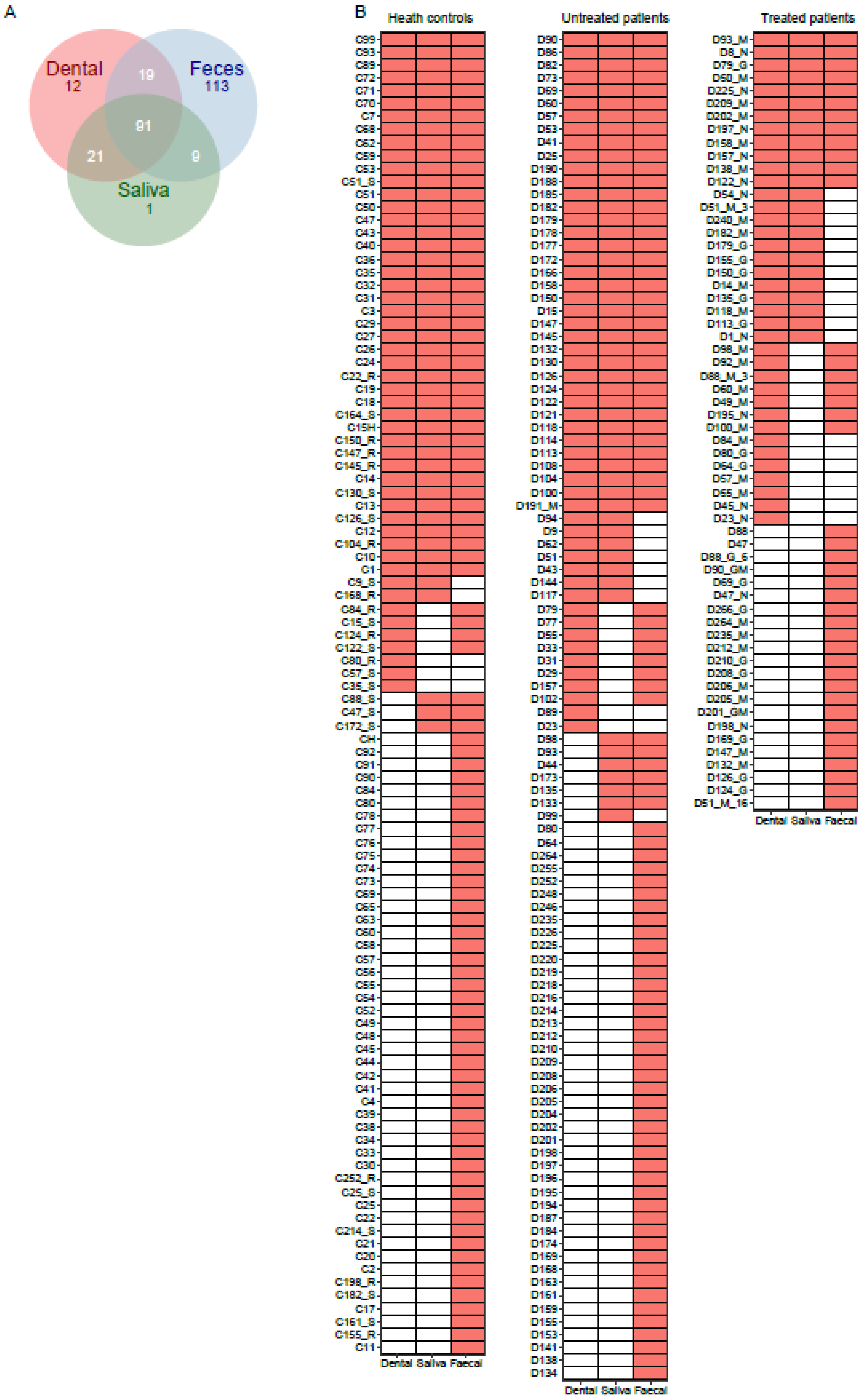

Figure S1| Sample collection. A, Overlap of volunteers between the dental plaque, saliva, and fecal samples. **B,** Available samples from each volunteer. Red tile indicated the available samples, and white tile indicated no available sample.

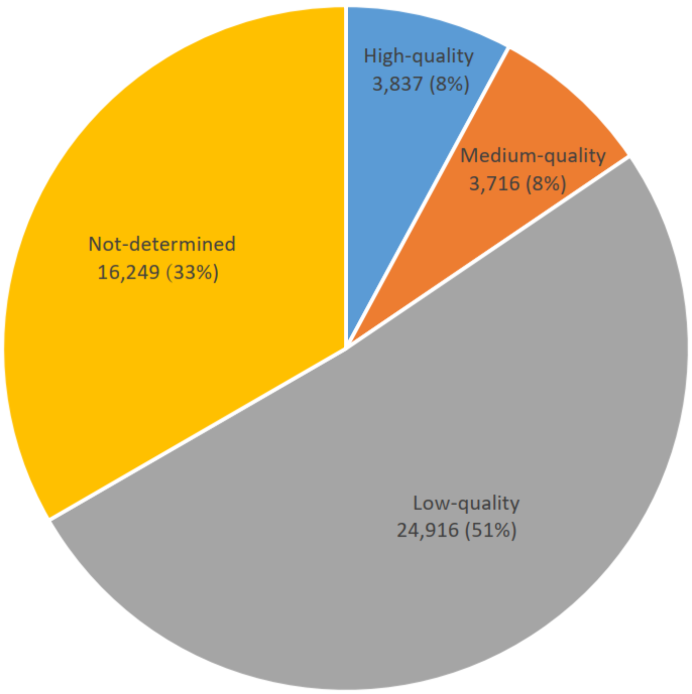

Figure S2| CheckV assessment of 48,718 vOTUs.

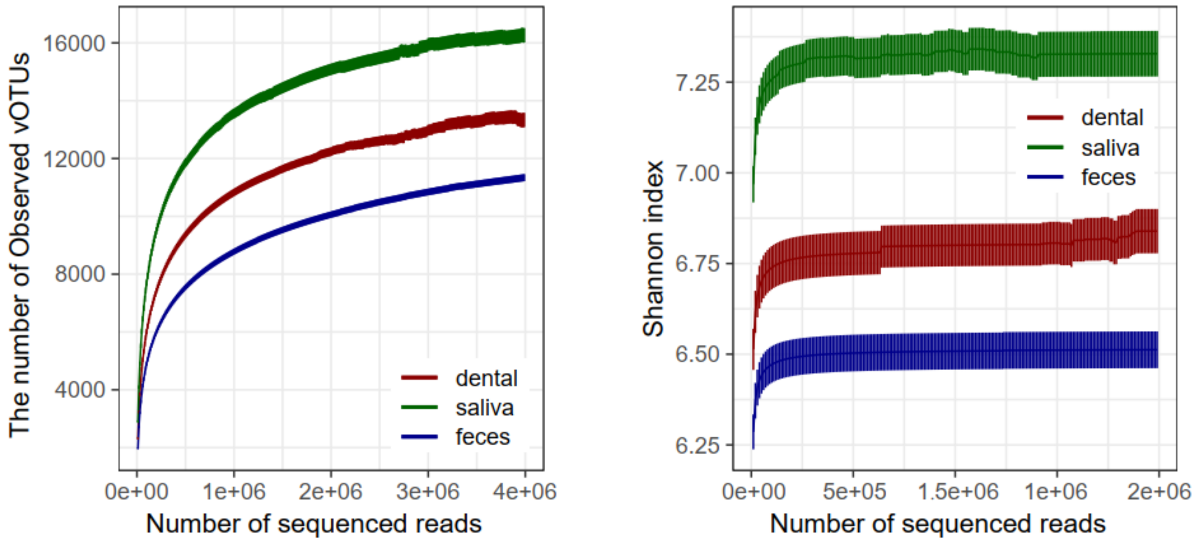

Figure S3| Rarefaction curves for the number of observed vOTUs and Shannon diversity index.

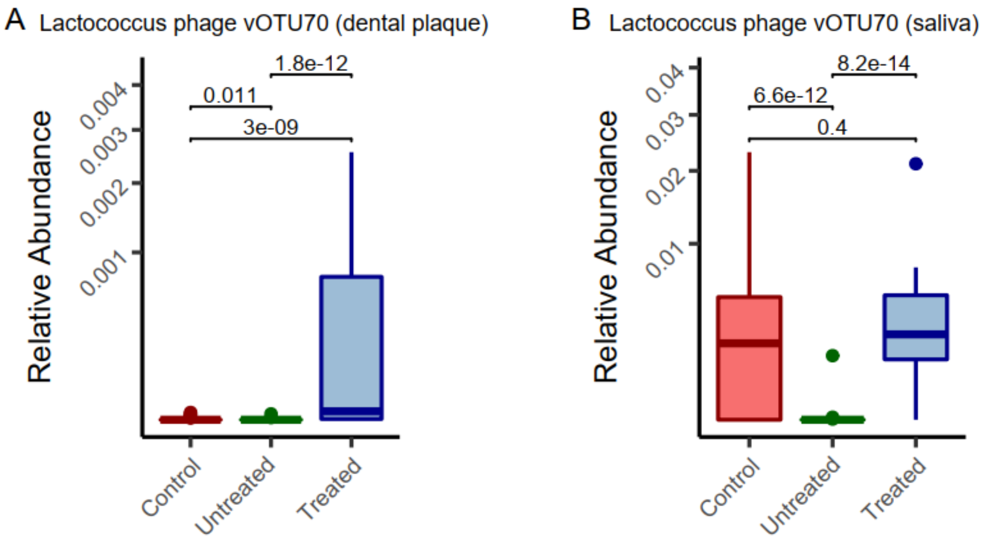

Figure S4| Comparison of relative abundance of *Lactococcus phage vOTU70* among healthy controls, untreated and treated patients with RA. A, Dental plaque samples. **B,** Saliva samples.

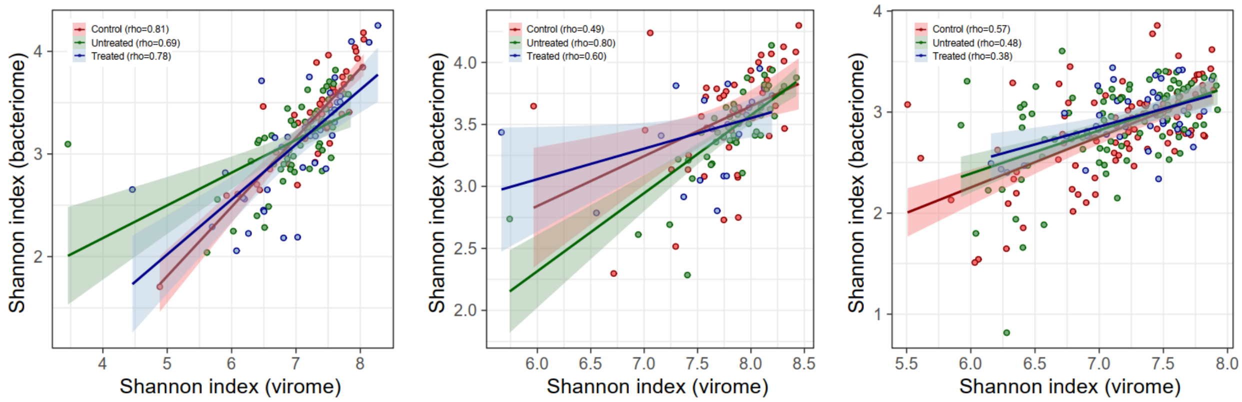

Figure S5| The correlation analysis of Shannon index between bacteriome and virome in oral and gut samples from healthy controls, untreated and treated patients with RA. Left plot, dental plaque. Center plot, saliva. Right plot, fecal samples.

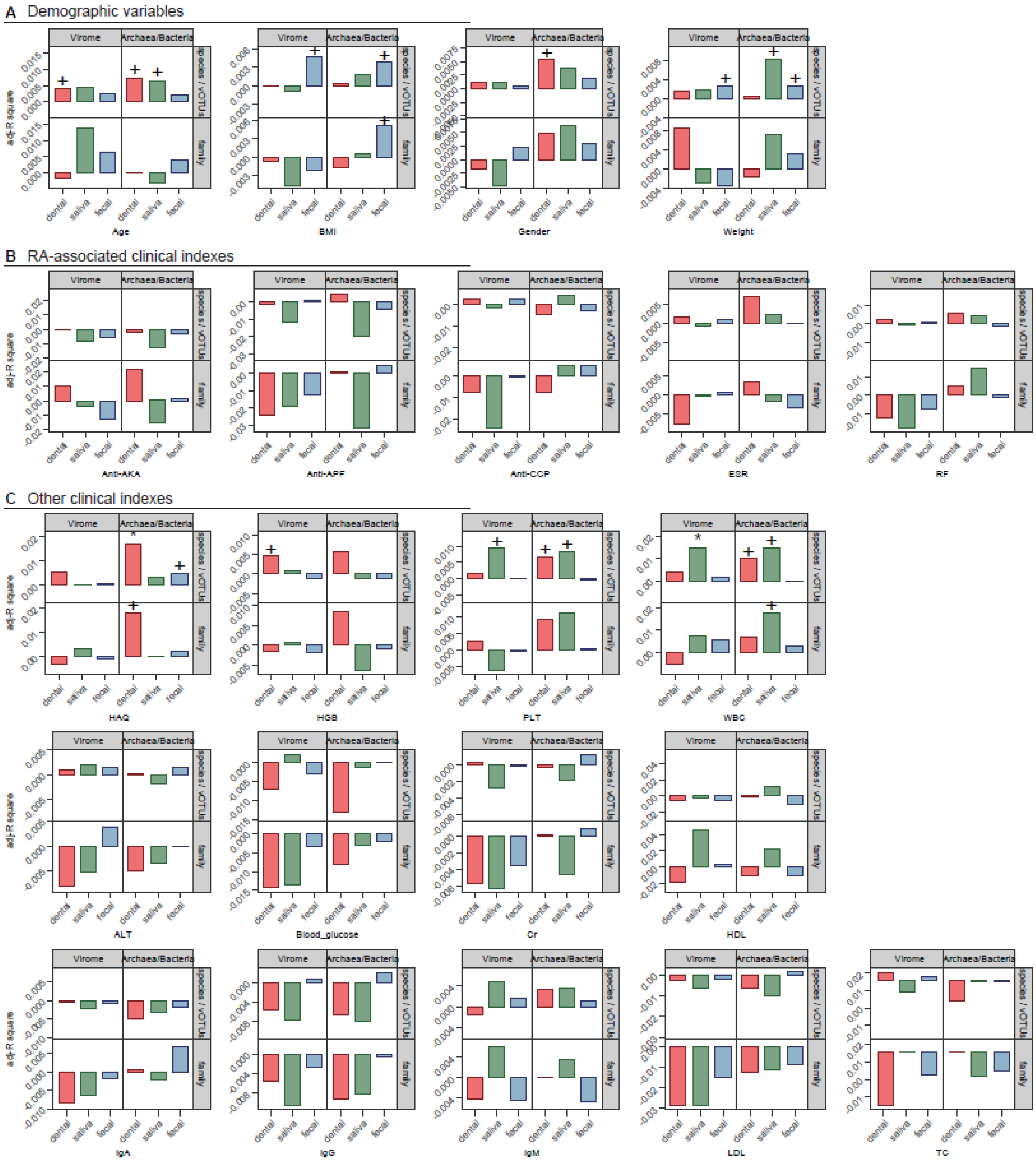

Figure S6| The effect size of clinical indexes on the oral and gut virome and bacteriome. *Adonis*, + adjusted P<0.05, * adjusted P<0.01.
